# Prescription Drug Diversion: Predictors of Illicit Acquisition and Redistribution in Three U.S. Metropolitan Areas

**DOI:** 10.3934/publichealth.2015.4.762

**Published:** 2015-12-02

**Authors:** Shana Harris, Valentina Nikulina, Camila Gelpí-Acosta, Cory Morton, Valerie Newsome, Alana Gunn, Heidi Hoefinger, Ross Aikins, Vivian Smith, Victoria Barry, Martin J. Downing

**Affiliations:** 1 Department of Anthropology, University of Central Florida, 4000 Central Florida Boulevard, Howard Phillips Hall 309, Orlando, FL 32816-1361, USA; 2 Psychology Department, Queens College, City University of New York, 6530 Kissena Boulevard, SB A344, Queens, NY 11367-1597, USA; 3 Social Science Department, LaGuardia Community College, City University of New York, 31-10 Thomson Avenue, Long Island City, NY 11101, USA; 4 School of Social Work, Rutgers University, 536 George Street, New Brunswick, NJ 08901, USA; 5 National Development and Research Institutes, 71 West 23rd Street, 4th Floor, New York, NY 10010, USA; 6 Department of Social Work, Binghamton University, P.O. Box 6000, Binghamton, NY 13902-6000; 7 Science Department, Berkeley College, 3 East 43 Street New York, NY 10017, USA; 8 Graduate School of Education, University of Pennsylvania, 3700 Walnut Street Philadelphia, PA 19104, USA; 9 Sociology and Criminology Department, Cabrini College, 610 King of Prussia Road, Radnor, PA 19087-3698, USA; 10 Department of Pediatrics, Harlem Hospital Center, 506 Malcolm X Boulevard, New York, NY 10037, USA; 11 Public Health Solutions, 40 Worth Street, 5th Floor, New York, NY 10013, USA

**Keywords:** prescription drugs, diversion, illicit behavior, Internet-based study, United States

## Abstract

**Objective:**

Prescription drug diversion, the transfer of prescription drugs from lawful to unlawful channels for distribution or use, is a problem in the United States. Despite the pervasiveness of diversion, there are gaps in the literature regarding characteristics of individuals who participate in the illicit trade of prescription drugs. This study examines a range of predictors (e.g., demographics, prescription insurance coverage, perceived risk associated with prescription drug diversion) of membership in three distinct diverter groups: individuals who illicitly acquire prescription drugs, those who redistribute them, and those who engage in both behaviors.

**Methods:**

Data were drawn from a cross-sectional Internet study (*N* = 846) of prescription drug use and diversion patterns in New York City, South Florida, and Washington, D.C.. Participants were classified into diversion categories based on their self-reported involvement in the trade of prescription drugs. Group differences in background characteristics of diverter groups were assessed by Chi-Square tests and followed up with multivariate logistic regressions.

**Results:**

While individuals in all diversion groups were more likely to be younger and have a licit prescription for any of the assessed drugs in the past year than those who did not divert, individuals who both acquire and redistribute are more likely to live in New York City, not have prescription insurance coverage, and perceive fewer legal risks of prescription drug diversion.

**Conclusion:**

Findings suggest that predictive characteristics vary according to diverter group.

## 1. Introduction

The nonmedical use of prescription drugs, use not intended by a prescribing physician [1], has been well documented in the United States [2]–[6]. There are as many as 6.8 million current nonmedical users of psychotherapeutic drugs (e.g., pain medications, stimulants, depressants) in the country; of those, 4.9 million use pain medications and 1.2 million use stimulants [7]. A potentially related problem of nonmedical prescription drug use is prescription drug abuse, which has increased markedly in recent years [1],[8]–[11]. Between 1998 and 2008, the incidence of prescription drug abuse increased by 400% [12]. Among young people, for instance, prescription drugs are the second most-abused category of drugs after marijuana [13]. As such, prescription drug abuse is the nation's fastest-growing drug problem [14].

Most experts consider prescription drug diversion to be a major driver of the country's prescription drug abuse epidemic [15]. “Diversion” is the transfer of prescription drugs from lawful to unlawful channels for distribution or use [16]. Diversion entails the illicit acquisition of drugs from medical sources, primarily through theft [17],[18], prescription forgery [19],[20], and “shopping” for lenient physicians or pharmacists [15],[21],[22]. It also entails the illicit acquisition of prescription drugs from nonmedical sources, usually through trading, stealing, borrowing, or purchasing from family and friends [23]–[28], street-level drug dealers or markets [16],[21],[29], and online pharmacies or websites [23],[30],[31]. In addition to acquisition, diversion includes the illicit redistribution of prescription drugs outside of a licit healthcare context [5],[20],[22],[25],[32]–[34]. As Inciardi and Cicero [35] noted, diversion — both acquisition and redistribution — can happen along all points in the prescription drug delivery process, from manufacturing site to wholesale distributor to physician's office to pharmacy to patient.

Recent research shows an increase in prescription drug diversion in the United States [5],[10],[35]. This is associated with a variety of issues, such as increased emergency room visits [5],[36], overdose and overdose-related deaths [9],[37]–[39], crime [10] [40], healthcare treatment and costs [5],[19],[41], and drug prevention and interception expenses [42]. Despite these concerns, diversion remains an understudied problem [11],[15],[16],[35],[43]. Most studies of prescription drug diversion focus on diversion behaviors among particular populations, especially college students [27],[28],[33],[44]–[47] and pain patients [25],[48],[49], or the diversion of specific prescription drugs, mainly opioids [26],[32],[50]–[52] and stimulants [24],[28],[53],[54]. Research with broader samples on more diverse drug classes would add to literature on prescription drug diversion.

Although illicit acquisition and redistribution of prescription drugs is a key contributor to prescription drug misuse [15],[25], there is a paucity of research examining correlates of engaging in illicit prescription drug acquisition and redistribution. The few studies that have studied predictors of prescription drug sharing and redistribution find that younger individuals are more likely to engage in the diversion of prescription drugs, including both acquisition and redistribution [55],[56]. Findings regarding gender are mixed; some studies find more diversion among women, including both acquisition and redistribution [56]–[59], while others report no gender differences in acquisition [60]. Race/ethnicity differences have also been observed; some studies find that White, non-Hispanic individuals are more likely to redistribute prescription drugs [56] while others report no race/ethnicity differences in acquisition and redistribution [61],[62]. Furthermore, the influence of income and prescription drug coverage on diversion behaviors [23] and perceptions of risks of diversion are understudied. A more in-depth understanding of correlates of diversion behaviors could elucidate pathways that contribute to misuse. Additionally, it is important to replicate results with new samples and recruitment methods, which could help clarify inconsistencies in previous findings.

The present study addresses this gap in the extant literature by offering insights into correlates of illicit acquisition and redistribution. Although both acquisition and redistribution are part of the prescription drug trade, these behaviors serve different functions and may be associated with different predictors that have not been previously examined in the prescription drug diversion literature. Drawing on studies of the illicit drug trade, it is likely that predictors of acquisition and redistribution are overlapping but also differ [63],[64]. The limited attention to heterogeneity of behaviors that comprise diversion could lead to erroneous assumptions about the characteristics of individuals involved in illicit diversion of prescription drugs. As described above, age, race/ethnicity, and gender differences [56]–[59] have been cited as predictors of diversion and anecdotal reports from medical providers and law enforcement suggest that diversion is more prominent among White, middle class communities [65]. However, current research evidence is insufficient to conclude whether these characteristics describe all individuals involved in prescription drug diversion or only those who acquire the drugs illicitly. Therefore, this study analyzes correlates of membership in three distinct diverter groups in these areas: 1) individuals who illicitly acquire prescription drugs, 2) individuals who redistribute them, and 3) individuals who engage in both behaviors. We analyze a number of variables that have been identified as potentially important correlates of diversion behaviors [23],[66] across these groups, including demographics, background characteristics, and perceptions of legal risk associated with drug diversion. We present data from a cross-sectional Internet study of prescription drug use and diversion patterns in three metropolitan areas with high prevalence rates of prescription drug diversion [5],[22],[29] in the eastern United States: New York City, South Florida, and Washington, D.C.

## 2. Methods

### 2.1. Participants

The sample consisted of 846 participants (248 males; average age = 36 years) drawn from the metropolitan areas of New York City (*n* = 348), South Florida (*n* = 202), and Washington, D.C. (*n* = 319). These cities were targeted for analysis because of previous documentation of prescription drug diversion in these metropolitan areas [5],[22],[29].

### 2.2. Procedure

The present study utilized online surveys to collect data from participants. All participants for this study were recruited through cost-free advertisement campaigns on Craigslist.org from May 2013 to April 2014. Craigslist is a publicly accessible, online bulletin board where users can post advertisements for real estate, employment, sexual encounters, volunteering, and other goods and services. It is a highly trafficked site, currently ranked 9th in the United States [67]. Past studies of drug use have successfully recruited participants for online research through this site [68]–[71].

The present study used elements of time-space sampling for its Craigslist advertisements [72]. A member of the research team posted an advertisement in the “volunteers” section of Craigslist at randomly selected hours. A true random number service (www.random.org) was used to randomly select the hour in which a daily post occurred. Recruitment occurred once per day from 7:00am to 12:00am. A total of 325 advertisements were posted during the recruitment period. Advertisements instructed interested persons to click on an embedded link to an online survey. These procedures are similar to those used in past studies [68],[73]. As in these other studies, the respondent sample may have been biased because the participants self selected.

Individuals who clicked on the survey link in the Craigslist advertisements were taken directly to the survey information and consent page, which included a description of study procedures and risks/benefits of participation. Participants were asked on the consent page to verify that they are at least 18 years of age. Once age was verified, participants were asked if they agree to participate in the study. Those who clicked “YES, I agree to participate” to the participation question were considered to have provided consent to participate and were advanced to the next screen, which started the survey. Only individuals who consented were eligible to participate. Those who consented to participate and completed the survey were given the option to provide an email address for entry into a drawing for one of five $100 Amazon gift cards.

To reduce the potential for multiple survey submissions by a single individual, the Internet Provider (IP) addresses of the participants were recorded through the survey host (Qualtrics). Although IP addresses do not specifically identify individuals, they are still considered a source of identifying information because they can be traced back to a computer with potentially one owner/user [69],[74]–[76]. To preserve confidentiality, all IP addresses were destroyed after checking for duplicate submissions. We identified 21 duplicate submissions, which were removed from data analyses. The first entry was retained as evidenced by an earlier timestamp associated with the IP address. Participant data were also removed from analyses because of extensive missing data (i.e., no more than 4 initial questions answered, *n* = 32) or because they failed to meet the study inclusion criteria by obtaining the link to the survey from sources other than Craigslist (*n* = 16) or by residing outside of the targeted geographical areas (*n* = 33). The study protocol received Institutional Review Board approval through the National Development and Research Institutes.

### 2.3. Measures

#### 2.3.1. Demographics and background characteristics

Demographics and background characteristics were collected for all participants. Seventy one percent of the sample identified as female. On average participants were 36 years of age [range 18 to 82 years]. Participants were asked to identify their race/ethnicity from the following list of categories: White/Caucasian (65%), African American/Black (13%), Hispanic/Latino (12%), Asian/Hawaiian/Pacific Islander (5%), American Indian/Alaskan Native (1%), more than one race (3%), and other (1%). Because 65% of the participants identified as White/Caucasian and no other group was overwhelmingly represented, participants were assigned to one of two groups: White (65%) and Non-White (35%). Participants were asked to report their annual income by choosing from one of nine categories: less than $10,000 (26%), $10,000 to $20,000 (15%), $20,001 to $30,000 (12%), $30,001 to $50,000 (17%), $50,001 to $70,000 (10%), $70,001 to $100,000 (14%), $100,001 to $150,000 (6%), $150,001 to $200,000 (1%), and more than $200,000 (1%). Because several income categories were too small to analyze, and to maximize analytical power, income categories were collapsed into three categories: below $30,000 (53%), $30,001 to $70,000 (26%), and above $70,000 (21%). Participants were asked to select their highest attained level of education from the following five categories: less than a high school degree (3%), high school/GED (14%), some college (37%), college degree (32%), and graduate/professional degree (15%). To maximize analytical power, education categories were collapsed into two: high school or below (16%) and some college and above (83%). Participants also reported if they currently have prescription drug insurance coverage (85%) and whether they had a licit prescription for opioids, stimulants, sedatives and tranquilizers (including anti-depressants), and sexual enhancement/erectile dysfunction drugs for a legitimated medical purpose within the past year (64%).

Participants also reported sources of illicitly-obtained drugs. Thirteen percent were obtained from medical sources, 32% were given by friends/family, 27% were purchased from friends/family, 15% were purchased from a dealer, 4% were stolen from friends/family, 4% were obtained online, and 4% were obtained from other sources. Participants reported that 47% of the redistributed prescription drugs were given away, 28% were traded, and 25% were sold.

#### 2.3.2. Illicit prescription drug diversion behaviors

Participants were asked about their illicit prescription diversion behavior over the last 12 months for four drug classes: opioids, stimulants, sedatives and tranquilizers (including anti-depressants), and sexual enhancement/erectile dysfunction drugs. Across all analyses, these four drug classes were collapsed into a single prescription drug category for analysis. Questions assessed whether participants acquired prescription drugs through various medical channels (e.g., doctor shopping, fabricated medical problem) or non-medical channels (e.g., family member, friend, drug dealer, online retailer) or redistributed any prescription drugs. Participants were classified into four mutually exclusive groups: 1) non-diverters (*n* = 521) representing individuals who did not report any involvement in illicitly acquiring or redistributing prescription drugs, 2) illicit acquirers (*n* = 145) representing individuals who reported illicitly acquiring prescription drugs, 3) illicit redistributors (*n* = 62) representing individuals who reported illicitly redistributing prescription drugs, and 4) those who were both illicit acquirers and redistributors (*n* = 118) representing individuals who reported both acquiring and redistributing prescription drugs illicitly.

#### 2.3.3. Perceptions of legal risks of prescription drug diversion

Participants were asked a series of items to elicit their perceptions of the legal consequences that people risk if they: (1) buy, sell, trade, or give away their prescription drugs; (2) buy, sell, trade, or give away prescription drugs that are not their own; (3) approach a doctor for prescription drugs for nonmedical use; (4) order prescription drugs not prescribed to them over the Internet; (5) smuggle prescription drugs from abroad; and (6) forge doctor prescriptions or call-in false prescriptions. Response options included no risk, slight risk, moderate risk, and great risk. Higher scores represented greater perceived risk of legal consequences. These items were averaged (i.e., for every participant his/her responses to each of the items were summed and divided by six, the number of items of the scale) to create an index score for perceived risks of prescription drug diversion (*a* = .87). The index score represents the average rating of risk that the participants attributed to engaging in diversion of prescription drugs. Because no gold standard assessment exists for measuring risk associated with diversion, the authors created this scale for the purposes of this study. Past research studying risk perceptions associated with drugs have generated similar instruments to answer their unique research questions [77]–[79].

#### 2.3.4. Perceptions of legal risks of illicit drug diversion

Two questions were asked to elicit participants' perceptions of the legal consequences that people risk if they: (1) buy, sell, trade, or give away marijuana and (2) buy, sell, trade, or give away other narcotics (e.g., cocaine, methamphetamine, heroin, etc.). These illicit drugs make up the highest percentage of the illicit drug market in the United States [80],[81]. Response options included no risk, slight risk, moderate risk, and great risk. Higher scores represented greater perceived risk of legal consequences. These items were averaged (i.e., for every participant his/her responses to each of the items were summed and divided by two, the number of items of the scale) to create an index score for perceived risks of illicit drug diversion (*a* = .70). Similar to the assessment of risk perceptions associated with diversion of prescription drugs, the authors created this scale for the purposes of this study.

### 2.4. Data analysis

To minimize the impact of missing data on the analyses, multiple imputations were conducted using SPSS [82]–[84]. This approach utilizes available data to impute missing values and is the preferred technique for handling missing data that minimizes bias. Multiple imputation is the preferred method for handling missing data which relies on participant's own observed values and random noise to generate missing values. This approach maintains the joint distribution between variables (e.g., variance, correlations) and minimizes the bias in estimates of missing values [83],[84]. No variable was missing more than 25% of data. As is standard with SPSS [82], there were four imputed datasets generated for missing values and the fifth one pooled these data together. Predictors with missing data were imputed based on other predictors or demographics in the dataset (age, area of residence, gender, race/ethnicity, education level, student status, annual income, prescription drug insurance coverage, receipt of a prescription in the last year, legal risk of prescription drug diversion, and legal risk of illegal drug diversion). Dependent variables were not imputed. Group differences between participants' diversion classification (i.e., non-diverter, illicit acquirer, illicit redistributor, and both illicit acquirer/redistributor) and demographic characteristics and risk perception variables were assessed with Chi-Square tests and Analyses of Variance (ANOVAs), followed up with planned linear contrasts. Linear contrasts first compared those not involved in illicit behavior to all three diverter groups, then compared the illicit acquirers/redistributors to the other two diverter groups and finally compared the illicit acquirer to the redistributor groups. Subsequently, multivariate logistic regressions were conducted to assess which demographic and risk perception variables remain correlated with illicit acquisition and redistribution (by participant diversion classification) when other correlates were controlled.

## 3. Results

### 3.1. Demographic and Background Characteristics

Table 1 shows the demographic and background characteristics for the sample. Assignment to diversion categories was associated with all of the predictors except for gender and education level (see Table 1). It was also associated with self-reported consumption of illicitly acquired prescription drugs. Of note, a higher percentage of participants in the illicit acquirer/redistributor group compared to the other groups were White, currently a student, had an income above $70,000 per year, did not have prescription drug insurance coverage but had a licit prescription for any class of drugs assessed in this study in the past year. This group was also younger than any other group with a mean age of 28.38 years. Furthermore, those in the illicit acquirer/redistributor group reported the lowest perceived legal risk associated with both prescription drug and illegal drug diversion. Additionally, when comparing only the three diverter groups on these demographics and background characteristics, the differences remained significant (Supplemental Table 1). Results from linear contrasts comparing mean differences in age and perceived legal risk of diversion in the four groups are presented in Supplemental Table 2.

### 3.2. Illicit Acquirer

Table 2 shows results from three separate regression analyses (one for each diversion classification group). For the acquirer group, age was negatively associated with illicit acquisition (*β* = −0.02, *OR* = 0.98, *p* < 0.05). Also, having a history of having a licit prescription for any class of drug assessed in this study was positively associated with illicit acquisition (*β* = 1.14, *OR* = 3.24, *p* < 0.001). Area of residence, race, gender, education, being a student, income, prescription insurance coverage, perceived legal risk of prescription diversion, and perceptions of legal risk of illicit drug diversion were not associated with membership in the illicit acquirer group.

### 3.3. Illicit Redistributor

For the redistributor group, regression analyses demonstrated that age was negatively associated with illicit redistribution (*β* = −0.04, *OR* = 0.97, *p* < 0.01). As with illicit acquisition, a history of having a licit prescription for any class of drug assessed in this study was positively associated with illicit redistribution (*β* = 0.89, *OR* = 2.44, *p* < 0.05). Additionally, perceived legal risks of prescription diversion was negatively associated with redistribution (*β* = −0.87, *OR* = 0.42, *p* < 0.01). Area of residence, race, gender, education, being a student, income, insurance coverage, and perceptions of legal risk of illicit drug diversion were not associated with membership in the illicit redistributor group.

**Table 1.** Demographics and background characteristics across groups of participants defined by their involvement in diversion

	Overall Sample *N =* 846 (%)	Not Involved in illicit behavior *n* = 521 (%)	Illicit Acquirer *n* = 145 (%)	Illicit Redistributor *n* = 62 (%)	Illicit Acquirer/redistributor *n* = 118 (%)	χ^2^(df)
**Area of residence**						47.5 (6)***
New York City	335 (39.7)	182 (35.0)	54 (37.2)	20 (32.7)	79 (66.9)	
South Florida	197 (23.3)	131 (24.1)	35 (24.1)	21 (34.4)	10 (8.5)	
Washington, D.C.	312 (37.0)	207 (39.8)	56 (38.6)	20 (32.7)	29 (24.5)	
**Gender**						6.4 (3)†
Male	248 (29.4)	166 (31.9)	43 (29.7)	14 (22.6)	25 (21.6)	
Female	354 (70.6)	354 (68.1)	102 (70.3)	48 (77.4)	91 (78.4)	
**Race/ethnicity**						15.7 (3)***
Non-White	293 (34.8)	192 (36.9)	60 (41.7)	17 (27.4)	24 (20.7)	
White	549 (65.2)	328 (63.1)	84 (58.3)	45 (72.6)	92 (79.3)	
**Education level**						2.6 (3)
H.S. or below	140 (16.6)	89 (17.1)	27 (18.9)	10 (16.1)	14 (11.9)	
Some college +	703 (83.4)	431 (82.9)	116 (81.1)	52 (83.9)	104 (88.1)	
**Student status**						64.8 (3)***
Student	249 (29.9)	125 (24.2)	40 (28.8)	13 (21.0)	71 (61.2)	
Not a student	585 (70.1)	392 (75.8)	99 (71.2)	49 (79.0)	45 (38.8)	
**Annual income**						60.4 (6)***
< 30,000	442 (52.7)	281 (54.5)	85 (59.0)	32 (53.3)	44 (37.3)	
30,000 to 70,000	222 (26.5)	150 (29.1)	40 (27.8)	13 (21.7)	19 (16.1)	
> 70,000	174 (20.8)	85 (16.5)	19 (13.2)	15 (25.0)	55 (46.6)	
**Rx insurance coverage**						127.5 (3) ***
Yes	599 (85.1)	390 (92.9)	107 (84.9)	47 (95.9)	55 (50.5)	
No	105 (14.9)	30 (7.1)	19 (15.1)	2 (4.1)	54 (49.5)	
**Rx in last year**						72.5 (3) ***
No	283 (36.1)	226 (47.2)	33 (24.3)	13 (24.1)	11 (9.6)	
Yes	501 (63.9)	253 (52.8)	103 (75.7)	41 (75.9)	104 (90.4)	
	*M* (*SD*)					*F* (*df_1_, df_2_*)
**Age**	36.16 (14.8)	38.58 (15.6)^a^	34.30 (13.3)^b^	35.03 (13.6)^b^	28.38 (9.1)^b^	29.6 (3,211)***
**Legal risk of Rx drug diversion**	3.19 (0.7)	3.37 (0.6)^a^	3.22 (0.8)^b^	3.04 (0.6)^b^	2.667 (0.6)^b^	39.6 (3,171.9)***
**Legal risk of illegal drug diversion**	3.20 (0.8)	3.38 (0.7)^a^	3.12 (0.9)^b^	3.21 (0.7)^b^	2.67 (0.6)^b^	34.6 (3,171.9)***

*Note:* χ^2^ and ANOVA analyses compared the demographic characteristics and perceptions of legal risk of drug diversion across the four groups (not involved, acquirer, redistributor, and acquirer/redistributor); Welch ANOVA results are presented to correct for heterogeneity of variance; † *p* < 0.10; * *p* < 0.05; ** *p* <.0 001; *** *p* < 0.001; All planned linear contrasts comparing group differences in age, and risk were significant at the *p* < 0.000 level. Superscripts indicate mean differences = same superscripts indicate no significant difference.

**Supplemental Table 1:** Demographics and background characteristics associations across three groups of participants involved in diversion

	Sample of Diverters *N =* 325 (%)	Illicit Acquirer *n* = 145 (%)	Illicit Redistributor *n* = 62 (%)	Illicit Acquirer/redistributor *n* = 118 (%)	χ^2^(df)
**Area of residence**					34.56 (6)***
New York City	153 (47.2)	54 (37.2)	20 (32.7)	79 (66.9)	
South Florida	66 (20.4)	35 (24.1)	21 (34.4)	10 (8.5)	
Washington, D.C.	105 (32.4)	56 (38.6)	20 (32.7)	29 (24.5)	
**Gender**					2.5 (2)
Male	82 (25.4)	43 (29.7)	14 (22.6)	25 (21.6)	
Female	241 (74.6)	102 (70.3)	48 (77.4)	91 (78.4)	
**Race/ethnicity**					13.7 (2)**
Non-White	101 (31.4)	60 (41.7)	17 (27.4)	24 (20.7)	
White	221 (68.6)	84 (58.3)	45 (72.6)	92 (79.3)	
**Education level**					2.4 (2)
H.S. or below	51 (15.8)	27 (18.9)	10 (16.1)	14 (11.9)	
Some college +	272 (84.2)	116 (81.1)	52 (83.9)	104 (88.1)	
**Student status**					38.5 (2)***
Student	124 (39.1)	40 (28.8)	13 (21.0)	71 (61.2)	
Not a student	193 (60.9)	99 (71.2)	49 (79.0)	45 (38.8)	
**Annual income**					36.7 (4)***
< 30,000	161 (50.0)	85 (59.0)	32 (53.3)	44 (37.3)	
30,000 to 70,000	72 (22.4)	40 (27.8)	13 (21.7)	19 (16.1)	
> 70,000	89 (27.6)	19 (13.2)	15 (25.0)	55 (46.6)	
**Rx insurance coverage**					25.4 (2) ***
Yes	209 (64.3)	107 (84.9)	47 (95.9)	55 (50.5)	
No	116 (35.7)	19 (15.1)	2 (4.1)	54 (49.5)	
**Rx in last year**					50.9 (2) ***
No	209 (73.6)	33 (24.3)	13 (24.1)	11 (9.6)	
Yes	75 (26.4)	103 (75.7)	41 (75.9)	104 (90.4)	
	*M* (*SD*)				*F* (*df_1_, df_2_*)
**Age**	32.3 (12.3)	34.30 (13.3)c	35.03 (13.6)^c^	28.38 (9.1)^b^	12.03 (2,153)***
**Legal risk of Rx drug diversion**	2.9 (0.7)	3.22 (0.8)^c^	3.04 (0.6)^c^	2.667 (0.6)^b^	14.3 (2,168.4)***
**Legal risk of illegal drug diversion**	3.0 (0.8)	3.12 (0.9)^c^	3.21 (0.7)^c^	2.67 (0.6)^b^	20 (2,178.7)***

*Note:* χ^2^ and ANOVA analyses compared the demographic characteristics and perceptions of legal risk of drug diversion across the four groups (not involved, acquirer, redistributor, and acquirer/redistributor); Welch ANOVA results are presented to correct for heterogeneity of variance; † *p* < 0.10; * *p* < 0.05; ** *p* < 0.001; *** *p* < 0.001; All planned linear contrasts comparing group differences in age, and risk were significant at the *p* < 0.000 level. Superscripts indicate mean differences = same superscripts indicate no significant difference.

**Table 2.** Multivariate logistic regressions predicting involvement in prescription drug diversion relative to non-diverters

	Acquirer			Redistributor			Acquirer/Redistributor		
	*B*	*SE B*	*OR* (*95CI*)	*B*	*SE B*	*OR* (*95CI*)	*B*	*SE B*	*OR* (*95CI*)
Area of residence (NYC reference) South Florida Washington, D.C.	−0.07	0.27	0.93 (0.53, 1.58)	0.60†	0.36	1.83 (0.90, 3.73)	−0.91*	0.43	0.40 (0.17,0 .93)
	−0.09	0.23	0.91 (0.58, 1.44)	−0.26	0.35	0.78 (0.39, 1.53)	−0.48	0.31	0.62 (0.34, 1.14)
White	−0.13	0.22	0.88 (0.58, 1.34)	0.42	0.34	1.53 (0.79, 2.90)	0.48	0.33	1.62 (0.85, 3.09)
Female	−0.06	0.22	0.94 (0.61, 1.46)	0.33	0.34	1.39 (0.72, 2.70)	−0.17	0.32	0.84 (0.45, 1.58)
Age	−0.02*	0.01	0.98 (0.96 , 1.0)	−0.04**	0.02	0.97 (0.94, .99)	−0.05***	0.01	0.95 (0.92, 0.98)
> High school	−0.09	0.28	0.91 (0.53, 1.56)	−0.06	0.41	0.95 (0.41, 2.13)	−0.58	0.40	0.56 (0.26, 1.23)
Student status	−0.04	0.27	0.96 (0.57, 1.63)	−0.33	0.39	0.72 (0.34, 1.54)	0.39	0.34	1.48 (0.75, 2.90)
Annual income (< 30,000, reference) 30,000 to 70,000 > 70,000	−0.04	0.24	0.96 (0.60, 1.44)	−0.31	0.37	0.74 (0.36, 1.52)	0.03	0.36	1.03 (0.51, 2.08)
	−0.18	0.33	0.83 (0.44, 1.60)	.073†	0.42	2.07 (0.90, 4.74)	0.71†	0.40	2.02 (0.93, 4.12)
Rx insurance coverage	−0.43	0.37	0.65 (0.31, 1.37)	0.17	0.65	1.19 (.30, 4.28)	−0.97*	0.41	0.38 (0.17, 0.85)
Rx in last year	1.14***	0.23	3.24 (2.00, 4.94)	0.89*	0.35	2.44 (1.22, 4.87)	1.86***	0.36	6.44 (3.16, 13.12)
Legal risk of Rx diversion	− 0.40†	0.20	0.67 (0.45, 1.00)	−0.87**	0.28	0.42 (−0.24, 0.72)	−0.71*	0.29	0.49 (0.28, 0.86)
Legal risk of illicit drug diversion	− 0.21	0.20	0.81 (0.54, 1.21)	0.17	0.28	1.18(0.69, 2.03)	−0.38	0.25	0.69 (0.42, 1.11)
*Note:* Results are based on the pooled imputed data; † *p* < 0.10; * *p* < 0.05; ** *p* < 0.0001; *** *p* < 0.001									

**Supplemental table 2:** Results from planned linear contrasts comparing means age, perceptions of legal risk associated with diversion of prescription and illicit drugs

	Age	Legal risk of Rx drug diversion	Legal risk of illegal drug diversion
	***t (df)***		
Contrast 1:			
Not involved in illicit trade compared to everyone else	5.7 (425.3)***	7.9(440.7)***	6.7(530.1)***
Contrast 2:			
Illicit acquirer/redistributor compared to illicit acquirer and illicit redistributor	−4.8 (218.9)***	−5.3(245.3)***	−6.3(238.2)***
Contrast 3:			
Illicit acquirer compared to illicit redistributor	−0.36 (112.8)	−0.13(135.2)	−0.95(154.0)
*Note:* Results are based on the pooled imputed data; *** *p* < 0.001			

### 3.4. Illicit Acquirer/Redistributor

Regression analyses demonstrated several main effects for individuals involved in both illicit acquisition and redistribution. As with the illicit acquisition and redistribution groups, age was negatively associated with both illicit behaviors (i.e., acquisition and redistribution) (*β* = − 0.05, *OR* = 0 .95, *p* < 0.001) and a history of having a licit prescription for any class of drug assessed in this study was positively associated with both illicit behaviors (*β* = 1.86, *OR* = 6.44, *p* < 0.001). However, there were some differences with the combined group. First, compared to participants in New York City (referent), participants in South Florida were less likely to participate in both illicit acquisition and redistribution of prescription drugs (*β* = − 0.91, *OR* = 0.40, *p* < 0.05). Second, perceived legal risks of prescription drug diversion was negatively associated with being in the combined illicit acquisition and redistribution group (*β* = − 0.71, *OR* = 0.49, *p* < 0.05). In fact, those who engaged in both illicit acquisition and redistribution had the lowest perception of legal risk associated with prescription and illegal drug diversion of all the groups assessed (see Table 1). Finally, participants who had insurance with prescription drug coverage were less likely to engage in both acquisition and redistribution than those without such coverage (*β* = − 0.97, *OR* = 0.38, *p* < 0.05). Race, gender, education, being a student, income, and perceptions of legal risk of illicit drug diversion were not associated with membership in the illicit acquirer/redistributor group.

## 4. Discussion

The present study assessed predictors of illicit prescription drug acquisition and redistribution using an Internet sample of individuals residing in three metropolitan areas in the United States. Relative to those not involved in diversion activities, illicit acquirers and illicit redistributors were more likely to be younger and have a licit prescription in the past year. Also, illicit redistributors perceived less risk associated with prescription drug diversion. These predictors were also significant for those engaged in both illicit activities (acquisition and redistribution). Individuals engaging in both activities were also more likely to live in New York City and not have insurance with prescription drug coverage. Additionally, while our results support previous research findings on illicit prescription drug behaviors being more prevalent among those who are young and White [56]–[59], our findings show other variables—largely unexplored in the literature—to be more significant in their positive association to the illicit acquisition and redistribution of prescription drugs.

Our findings add to the literature on the association between diversion behaviors and income, prescription drug insurance coverage, and having a licit prescription. These topics have received little attention in past research [23]. This study corroborated that having a prescription, and simultaneous lack of prescription drug coverage, were more important in explaining engagement in both illicit acquisition and redistribution than income. These findings may suggest that individuals without prescription drug coverage are more likely to engage in the illicit trade of prescription drugs. The negative consequences associated with diversion of prescription drugs [9],[37]–[39] generated calls for stricter prescription drug policies that would reduce the availability of certain medications such as prescription opioids. The present study suggests that, in addition to prescription practices, policy makers should consider prescription drug insurance coverage as a variable when examining prescription drug diversion and prevention. Arguably, when uninsured, individuals may resort to alternative (illicit) ways of acquiring prescription drugs, whether out of “medical need” or not. This is a largely unexplored theme, and more research is needed to understand the extent of this phenomenon. Nonetheless, it would be interesting to examine how participation in illicit redistribution and acquisition is impacted by the Affordable Care Act in the United States, which has enabled more Americans to obtain healthcare coverage but has not necessarily translated into prescription drug coverage.

The study's findings suggest that there are differences in the characteristics of individuals who acquire prescription drugs illicitly as compared to those who redistribute them and those who engage in both of these activities. As in previous literature on individuals involved in the illicit drug trade [63],[64], predictors such as younger age and having legal access to prescription drugs in the past year describe all individuals involved in diversion behaviors in the study. Notably, those who redistribute prescription drugs perceive themselves to be less at risk of legal consequences related to their actions. As described in other literature [66], this may support the notion that lower perceptions of legal consequences may contribute to the acceptance of diversion and drug use behaviors. Although causal conclusions cannot be drawn from this correlational study, from a prevention perspective, educating the public about the illegality of drug diversion should be considered in future studies as a possible diversion deterrent. Future studies could also help further examine the motivations behind illicit acquisition and redistribution. Qualitative research of individuals who are involved in prescription drug diversion could help better understand such motivations [54] and lead to targeted interventions that could help discourage diversion [23].

The present findings partially replicate past research on the association of demographic variables with diversion. For instance, the rates of illicit acquisition and redistribution in our sample with 22% reporting acquiring and 31% reporting redistributing were comparable to those of previous studies [23]. Furthermore, in this sample ranging in age from 18–82, the younger the participant was, the more likely they were to acquire and redistribute prescription drugs illicitly [24],[50],[85]. Recent evidence suggests that younger individuals (adolescents and young adults) calculate risk differently from older individuals [86], and future research should consider how the development of risk-taking perceptions and behaviors contributes to diversion. These findings also highlight the importance of targeting prevention and intervention strategies towards younger adults. In particular, because this study was conducted with Internet users, online and social media forums may be relevant platforms to reach younger individuals.

In our examinations of group differences, White individuals were more likely to engage in illicit redistribution or both acquisition and redistribution but not solely acquisition of prescription drugs. These race/ethnicity differences were reported in some previous studies [56] but not others [60],[62]. It is important to note, however, that although groups differed by race/ethnicity, this variable was not a significant predictor of membership in diversion categories in multivariate analyses. This suggests that other variables may be more important factors to examine when studying diversion behaviors. We also did not find that diversion behaviors differed by gender, although some past studies have found such differences [39],[58],[87],[88]. For example, gender differences were observed in one study of in-person surveys conducted in public spaces [58]. It is likely that the discrepant findings are driven by the different methods used across studies, and are limited in this study in part because the sample was primarily female and gender was therefore not equally represented across diversion groups. The online survey method utilized in the present study may have contributed to reduced social desirability bias and may have elicited more honest responses than in-person interviews across a range of participants [89],[90].

An examination of group differences among diverter groups suggests furthermore that individuals engaging in both illicit acquisition and redistribution are more likely to reside in the New York City metropolitan area, be younger, be White, be students, be wealthier, and have licit prescriptions but no insurance coverage. They are also less likely to associate negative consequences with engaging in the illicit trade of prescription drugs. Although not all of these variables significantly predicted membership in the acquirer/diverter group in multivariate analyses, suggesting that differences in diversion behaviors may be driven by fewer variables, examination of diverter groups suggests that the acquirer/diverter group may have a unique profile as compared to other groups. Some of the characteristics of this profile, such as student status and being White, overlap with characteristics of individuals who are likely to engage in the trade of stimulant prescription drugs [54]. This could be a reflection of increasing prescription drug use and availability, for example, with stimulants in collegiate populations, as documented by other researchers [2],[4],[27],[33],[46]–[48]. However, the present findings are based on a sample of individuals who divert a range of prescription drugs and not solely stimulants. Overall, the findings suggest that individuals who engage in both acquisition and redistribution may be different from non-diverters and those who acquire or redistribute illicitly. This group that engages in both diversion behaviors should be examined more thoroughly in future studies.

There are some limitations to the present study. The online recruitment of participants may have yielded a sample of individuals that does not fully represent the general population or the general substance abusing population in the United States [91]. Many demographic characteristics of the present sample do, however, corroborate extant research. For example, drug use tends to peak at an earlier age, and the population of drug users is more male dominated. Previous research also suggests that individuals with regular access to the Internet tend to be younger, White adults of higher socioeconomic status [92],[93]. Furthermore, recent evidence suggests that the demographics of Internet users in the United States have become more representative of the general population [94],[95]. We are unaware of any research that specifically examines demographics of Craigslist users across U.S. metropolitan areas. Additionally, it is possible that those who engaged in both illicit acquisition and redistribution were more likely to take our survey because of self-selection in responding to an online research solicitation for a topic of personal relevance. Despite this possibility, the sub-sample of illicit redistributors was relatively small, limiting the strength of findings both within this group and across the sample. Future studies might find more effective strategies for Internet-based recruitment to garner a wider and more representative sample through the use of other websites or online platforms.

Another limitation of this study is that legal perceptions of prescription diversion risk were conceptualized as a singular category. In other words, legal risks associated with different behaviors (i.e., buying, selling, trading, or giving away of prescription drugs) were not parsed out. Future studies could improve upon the content validity regarding this line of inquiry by asking about these diversion practices separately. Furthermore, subsequent diversion research that aims to better understand the perceived risks of these various practices should inform the design of more nuanced interventions.

A particular strength of the present study is the use of the online survey approach to data collection, which may have minimized social desirability bias and has been shown to be useful in recruiting high risk samples. Online surveys are an efficient, cost-effective, and increasingly common research method [96] and are particularly useful for collecting data from high risk or stigmatized populations (e.g., drug users, sexual minorities) [89],[97]–[101]. Some research also suggests that response rates of online surveys may be comparable to or higher than those for traditional survey methods such as phone or mail [100],[102],[103]. Furthermore, this type of research removes potential interviewer effects (i.e., social desirability) that can occur when attempting to collect data on sensitive topics during interviews [89],[90]. Indeed, at least one study corroborated the validity of this method, which noted a higher reporting of substance use behaviors via a web-based survey compared to phone or mail [104].

Another strength of our study is that much of the previous research on prescription drug diversion has focused on specific groups (e.g., college students, pain patients) or specific drugs (e.g., opioids, stimulants). By not limiting our study to a specific population or drug class, our study was able to conduct analyses across demographic groups and drug class. These findings are an important addition to the literature, as they show that various behaviors under the umbrella of drug diversion may differ across the wider population. Our findings also indicate that predictors of drug diversion vary across diversion behaviors. While this study does not explore motivations behind different diversion behaviors, future research on such motivations would better inform interventions for the prevention or mitigation of risk associated with these behaviors. Future research should also examine possible predictors of illicit acquisition and redistribution with respect to different drug classes. Inter-group comparisons of these behaviors would also help us better understand the motivators and pathways that drive prescription drug diversion in the United States.
